# Validation of the revised Oxford classification for IgA nephropathy considering treatment with corticosteroids/immunosuppressors

**DOI:** 10.1038/s41598-020-68087-y

**Published:** 2020-07-07

**Authors:** Takahito Moriyama, Kazunori Karasawa, Yoei Miyabe, Kenichi Akiyama, Shota Ogura, Tomo Takabe, Naoko Sugiura, Momoko Seki, Yuko Iwabuchi, Keiko Uchida, Kosaku Nitta

**Affiliations:** Department of Nephrology, Tokyo Women’s Medical University, 8-1 Kawada-cho, Shinjuku-ku, Tokyo, 162-8666 Japan

**Keywords:** Medical research, Nephrology

## Abstract

The Oxford classification for IgA nephropathy (IgAN) was updated in 2017. We have validated the revised Oxford classification considering treatment with corticosteroids/immunosuppressors. In this retrospective analysis, 871 IgAN patients were enrolled. Patients were divided into two groups, those treated with or without corticosteroids/immunosuppressors. The 20-year renal prognosis up to end-stage renal disease was assessed using the Oxford classification. In all patients, the renal survival rate was 87.5% at 10 years and 72.6% at 20 years. The T score alone was significantly related to renal prognosis in the Kaplan–Meier analysis and multivariate Cox regression analysis. In the non-treatment group (n = 445), E, S, T, and C scores were significantly related to renal survival rates, however, in the treatment group (n = 426), T score alone was significantly related to renal prognosis on Kaplan–Meier analysis, indicating that corticosteroids/immunosuppressors improved renal prognosis in E1, S1, and C1. In patients with E1, S1, or C1, the treatment group showed significantly better renal prognosis than the non-treatment group in univariate and multivariate analysis. The Oxford classification and T score were used to determine renal prognosis in IgAN patients. Corticosteroids/immunosuppressors improved renal prognosis, especially E1, S1, and C1 scores.

## Introduction

IgA nephropathy (IgAN) was first reported 50 years ago by Berger^1^. IgAN was initially labelled as a benign disease; however, it was later shown to have a poor long-term prognosis^2–5^. Although the prognostic risk factors of IgAN have not been clearly defined, hypertension, deterioration of renal function, and increased levels of proteinuria are known prognostic factors^2–5^. Histological findings may also inform prognosis^6–9^, although these factors have not achieved worldwide acceptance.

In 2009, the Oxford classification was reported by the International IgAN Network and International Renal Pathology Society^10,11^. In the Oxford classification, mesangial hypercellularity (M), segmental glomerulosclerosis (S), tubular atrophy/interstitial fibrosis (T), were selected as prognostic factors and endothelial hypercellularity (E) were selected as reactive factors against corticosteroids/immunosuppressors (MEST score). After this report of the Oxford classification, several validation studies were performed, with different results among those studies^12–25^. Using a multivariate analysis, it was determined that the T score was the most valuable marker of progression; however, other factors differed according to the clinical background (race, age), inclusion criteria (estimated glomerular filtration rate [eGFR] > 30 mL/min/1.73 m^2^, proteinuria > 0.5 g/day, minimum follow-up > 1 year), duration of follow-up, treatment, and endpoint of each study (eGFR slope, 50% reduction of eGFR, or end-stage renal disease [ESRD]). Interestingly, several reports validated not only the MEST score but also the crescent formation (as the C score), and multivariate analysis indicated that the C score was an independent factor for progression^12,17,18^. One meta-analysis of 16 validation studies with 2,893 patients confirmed that the M, S, T, and C scores were strongly related to renal prognosis^26^. Considering those reports and the previous exclusion criterion of eGFR < 30 mL/min/1.73 m^2^, which meant excluding rapid progressive cases, the MEST score was improved when cellular crescent and fibrocellular crescent formations (MEST-C score) were included^27^. The C score was defined as C0 (no crescents), C1 (crescent in > 0% but < 25% of glomeruli), and C2 (crescents in at least 25% of glomeruli). Corticosteroids/immunosuppressors improved the prognosis of patients with C1 lesions but not of those with C2 lesions; therefore, corticosteroids/immunosuppressors were recommended for treating IgAN patients with C1 lesions^18,27^.

It is important to note, however, that validation studies of the MEST score have generally excluded patients with rapidly progressing IgAN, defined by an eGFR < 30 mL/min/1.73 m^2^. Moreover, validation data on the revised Oxford classification are lacking. Accordingly, our aim in this study was to validate the revised Oxford classification (MEST-C score) among patients with IgAN, confirmed by renal biopsy.

## Results

### Clinical and histological findings, initial treatment, and prognosis in all IgAN patients

The study group included 871 patients with IgAN, who had > 8 glomeruli and were observed over a period of ≥ 1 year. The renal prognosis was evaluated using the revised Oxford classification and compared between patients treated with and without corticosteroids/immunosuppressants. The baseline data of all patients are shown in Table 1a. The median age was 31.0 years, and there were 356 (40.9%) male and 515 (59.1%) female patients. The median systolic blood pressure (SBP) was 120.0 mmHg, and the median diastolic blood pressure (DBP) was 74.0 mmHg. The median duration of follow-up was 8.0 years. Regarding the laboratory findings, the median eGFR was 77.0 mL/min/1.73 m^2^, and the median urinary protein excretion (U-Prot) was 0.68 g/day. Notably, in our study group, 11 patients (1.2%) had an eGFR < 30 mL/min/1.73 m^2^, with 323 patients (37.0%) having a U-Prot level < 0.5 g/day. Histological findings were as follows: 49.4% had M1, 44.9% had E1, 72.0% had S1, 21.7/5.9% had T1/T2, and 45.3/5.3% had C1/C2. Several major treatments for IgAN were started within 1 year after renal biopsy as the initial treatment (Table 1b); 426 patients (48.9%) were treated with corticosteroids/immunosuppressors. Among those 426 patients, 424 patients were treated with corticosteroids alone, and 13 patients were treated with corticosteroids and/or other immunosuppressive agents. One-hundred-and-ninety-two (22.0%) patients underwent tonsillectomy, 293 (33.6%) were treated with renin-angiotensin system (RAS) inhibitors, and 177 (20.3%) were treated with fish oil (Table 1b). One-hundred-and-fifteen patients (13.2%) progressed to ESRD during the follow-up period, and five patients died before reaching ESRD.

**Table 1:** Baseline characteristics of all 871 patients.

Baseline data	Unit	Values
(a)		
**Clinical findings**		
Age	Years	31.0 (24.0–41.0)
Pediatrics subjects (< 18 years)	%(n)	2.2 (19)
Sex	Male/female	356/515
BMI	kg/m^2^	21.3 (19.6–23.5)
SBP	mmHg	120.0 (110–132.0)
DBP	mmHg	74.0 (66.0–83.0)
MAP	mmHg	89.7 (81.3–99.0)
Duration of follow up	Years	8.0 (4.0–14.5)
**Laboratory findings**		
TP	g/dL	6.8 (6.3–7.2)
Cr	mg/dL	0.79 (0.67–1.00)
eGFR < 30 mL/min/1.73 m^2^	ml/min/1.73m^2^ % (n)	77.0 (60.0–95.6) 1.2 (11)
UA	mg/dL	5.5 (4.5–6.7)
T-Cho	mg/dL	192.0 (168.0–225.0)
TG	mg/dL	100.0 (73.0–144.0)
U-prot < 0.5 g/day	g/day % (n)	0.68 (0.3–1.4) 37 (323)
U-RBC (5 < , 5–25, 26–49, 50–99, 100 ≦)	counts/HPF	85, 365, 140, 119, 157
**Histological findings**		
M0/M1		441/430 (59.6/49.4%)
E0/E1		479/391 (55.1/44.9%)
S0/S1		243/628 (28.0/72.0%)
T0/T1/T2		631/189/51 (72.5/21.7/5.9%)
C0/C1/C2		454/370/46 (50.4/45.4/5.3%)
(b)		
**Initial treatment **		
Corticosteroids/immunosuppressors	426 (48.9%)	
Corticosteroids	424	
Immunosuppressors	13	
Mizoribine	8	
Calcineurin inhibitors	3	
Cyclophosphamide	1	
Tonsillectomy	192 (22.0%)	
RAS inhibitors	293 (33.6%)	
Fish oil	177 (20.3%)	
**Outcome**		
ESRD	115 (13.2%)	
Died before ESRD	5 (0.6%)	

*BMI* body mass index, *SBP* systolic blood pressure, *DBP* diastolic blood pressure, *MAP* mean arterial pressure, *TP* serum total protein, *Cr* serum creatinine, *eGFR* estimated glomerular filtration rate, *UA* serum uric acid, *T-cho* serum total cholesterol, *TG* triglyceride, *U-Prot* urinary protein excretion, *U-RBC* urinary red blood cells, *HPF* high power field, *M* mesangial hypercellularity, *E* endocapillary hypercellularity, *S* segmental sclerosis, *T* interstitial fibrosis/tubular atrophy, *C* crescents, *RAS* renin angiotensin systems, *ESRD* end stage renal disease.

**Figure 1:**
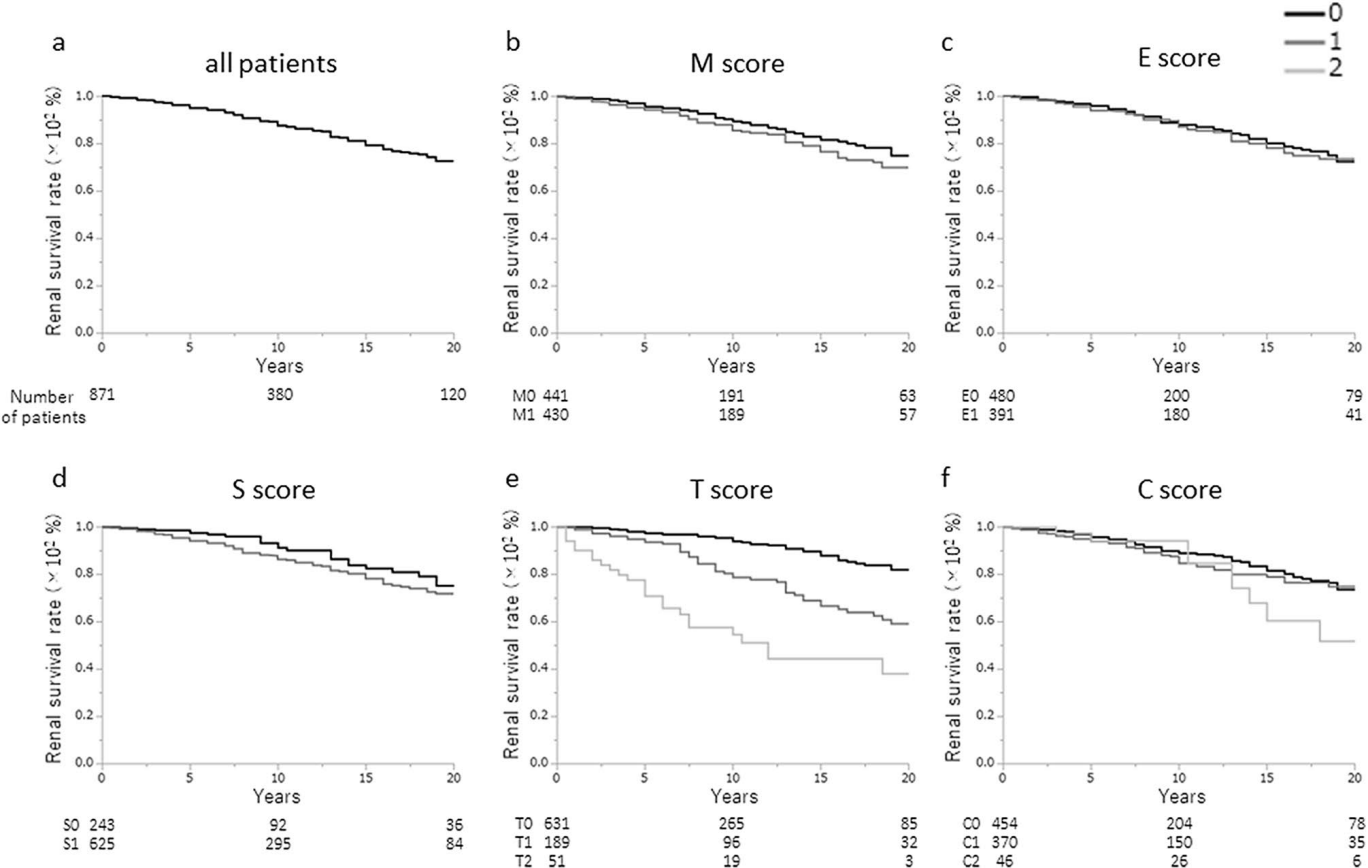
Renal survival rates of all patients. **(a)** The renal survival rate was 87.5% at 10 years and 72.6% at 20 years. Survival rates were similar between the following histological categories of the Oxford classification: **(b)** M0 (75.1%) and M1 (69.9%), p = 0.1100), **(c)** E0 (72.3%) and E1 (73.5%), p = 0.7055; and **(d)** S0 (75.2%) and S1 (71.7%), p = 0.0955. **(e)** The 20-year renal survival rate was significantly different across the T-score categories (T0, 82.1%; T1, 59.1%; and T2, 38.0%; p < 0.0001) but not **(f)** the C-score categories (C0, 73.4%; C1, 74.9%; and C2, 51.8%; p = 0.3067).

The 10-year renal survival rate was 87.5%, and the 20-year renal survival rate was 72.6% (Fig. 1a). There were significant differences in the 20-year renal survival rates for each Oxford classification based on the T score (T0, 82.1%; T1, 59.1%; T2, 38.0%; p < 0.0001), but not based on the M, E, S, or C scores (Fig. 1b–f).

### Clinical and histological findings and prognosis in the treatment and non-treatment groups

**Figure 2:**
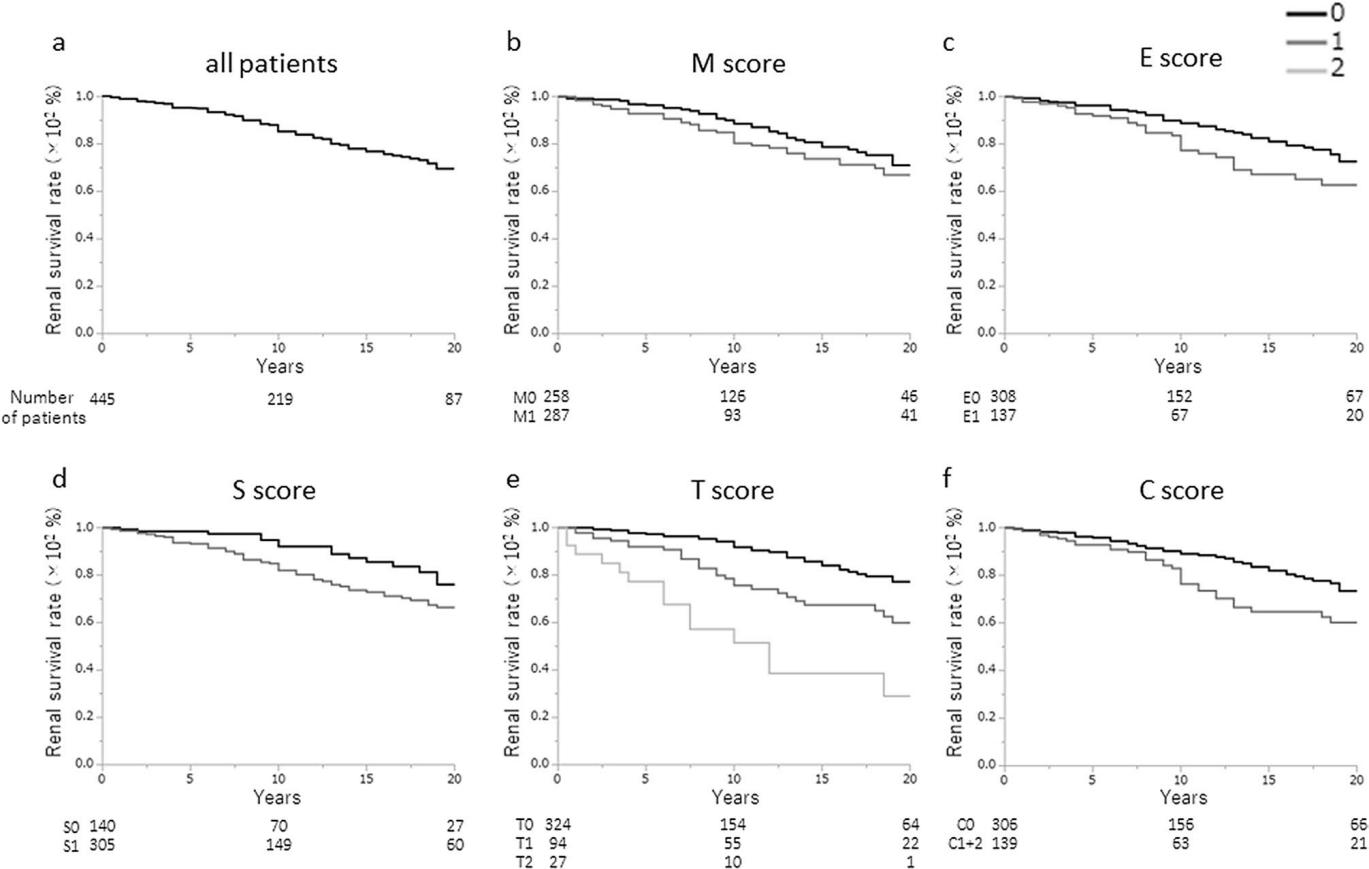
Renal survival rates of the non-treatment group. **(a)** The renal survival rate for the non-treatment group was 85.1% at 10 years and 69.4% at 20 years. Survival rates were similar between the Oxford **(b)** M0 (71.1%) and M1 (66.8%) categories (p = 0.1583), but significantly higher for **(c)** the E0 (72.6%) than E1 (62.7%) categories (p = 0.0222) and **(d)** S0 (76.0%) and S1 (66.4%) categories (p = 0.0219). **(e)** The 20-year renal survival rates were 77.3% for T0, 60.0% for T1, and 29.0% for T2, which were significantly different among the three groups (p < 0.0001). The renal survival rate was significantly higher for T0 than for either T1 (p = 0.0009) or T2 (p < 0.0001). The renal survival rate was significantly higher for T1 than T2 (p = 0.0019). **(f)** The 20-year renal survival rate was significantly higher for C0 than for C1 + C2 (C0, 73.5%; C1 + C2, 60.3%; p = 0.0228). Note that as there were only 5 patients in the C2 category, this group was combined with the C1 group.

**Figure 3:**
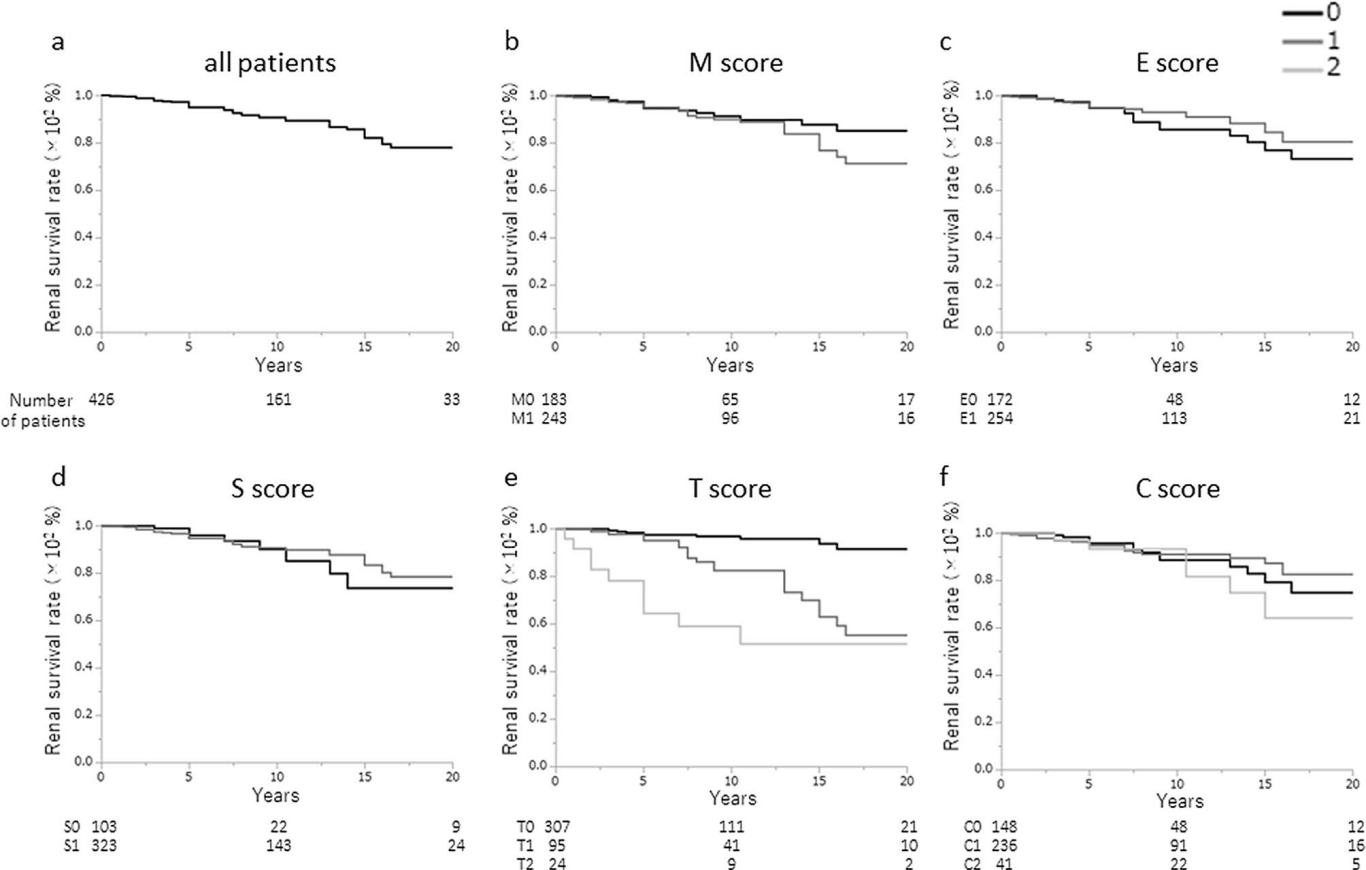
Renal survival rates of the treatment group. **(a)** The renal survival rate of treatment group was 90.6% at 10 years and 78.0% at 20 years, and they were similar for the **(b)** M0 (83.0%) and M1 (70.5%) categories (p = 0.2125), **(c)** E0 (73.4%) and E1 (80.5%) categories (p = 0.8183) and **(d)** S0 (73.8%) and S1 (78.4%) categories (p = 0.9111). **(e)** The 20-year renal survival rates were 90.6% for T0, 55.4% for T1, and 51.7% for T2, which were significantly different among the three groups (p < 0.0001). The renal survival rate was significantly higher for T0 than T1 (p < 0.0001) or T2 (p < 0.0001). The renal survival rate for T1 was significantly higher than for T2 (p = 0.0165). **(f)** The 20-year renal survival rate was not significantly different among the three C-score groups (C0, 74.9%; C1, 82.6%; C2, 64.2%; p = 0.4954). The renal survival rate for the C0 category was similar to that for C1 (p = 0.6672) and for C2 (p = 0.4924). The renal survival rate for C1 was similar to that for C2 (p = 0.2219).

The Oxford baseline data of the treatment group and the non-treatment group is shown in Table 2, as well as comparisons between the data in the two groups (Table 2). There were significant differences in the median SBP (p = 0.0473), total protein (TP) (p < 0.0001), eGFR (p = 0.0133), T-Cho (p < 0.0001), U-Prot (p < 0.0001), and U-RBC (p = 0.0045) between the groups. Regarding the histological findings used to determine the Oxford classification, M1, E1, S1, and C1/2 were found significantly more often in the treatment group than in the non-treatment group (M1: 57.0 vs. 42.0%, p < 0.0001; E1: 59.6 vs. 30.8%, P < 0.0001; S1: 75.8 vs. 68.5%, p = 0.0164; C1/C2: 55.4/9.6 vs. 30.1/1.1%, p < 0.0001); however, T1 and T2 were similar in both groups. The renal survival rate in the non-treatment group was 85.1% at 10 years and 69.4% at 20 years (Fig. 2a). There were significant differences in the renal survival rate based on E scores (E0, 72.6%; E1, 62.7%; p = 0.0222), S scores (S0, 76.0%; S1, 66.4%; p = 0.0219), T scores (T0, 77.3%; T1, 60.0%; T2, 29.0%; p < 0.0001), and C scores (C0, 73.5%; C1 + C2, 60.3%; p = 0.0075), but not in those based on M score (Fig. 2b–f). The renal survival rate of the treatment group was 90.6% at 10 years and 78.0% at 20 years (Fig. 3a). There were only significant differences between the renal survival rate based on the T score (T0, 90.6%; T1, 55.4%; T2, 51.7%; p < 0.0001). Interestingly, the renal survival rate based on E1, S1, and C1 increased more than that based on E0, S0, and C0, respectively, when corticosteroids/immunosuppressors were used as treatment (E0, 73.4%, E1, 80.5%, p = 0.8183) (S0, 73.8%, S1, 78.4, p = 0.9111) (C0, 74.9%; C1, 82.6%; p = 0.6672); however, these increases were not significant, and moreover, the renal survival rate based on C2 was still low (64.2%), despite treatment (Fig. 3b-f).

**Table 2:** Comparison of baseline characteristics between patients with or without corticosteroids/immunosuppressors.

Baseline data	Unit	Non-treatment group	Treatment group	P-value
**Clinical findings**				
Age	Years	31.0 (24.0–41.0)	30.0 (24.0–41.0)	0.8199
Sex	Male/female	174/271	182/244	0.2771
BMI	kg/m^2^	21.3 (19.6–23.3)	21.4 (19.6–23.7)	0.9953
SBP	mmHg	120.0 (110.0–132.0)	118.0 (110.0–130.0)	0.0473
DBP	mmHg	75.0 (66.0–84.0)	74.0 (66.0–82.0)	0.4872
MAP	mmHg	90.0 (80.8–100.0)	88.3 (81.5–98.0)	0.2391
**Laboratory findings**				
TP	g/dL	6.9 (6.5–7.3)	6.7 (6.2–7.1)	< 0.0001
Cr	mg/dl	0.80 (0.69–1.07)	0.78 (0.66–0.98)	0.1492
eGFR	ml/min/1.73 m^2^	73.3 (59.1–93.2)	79.8 (63.1–96.9)	0.0133
UA	mg/dl	5.4 (4.4–6.7)	5.6 (4.7–6.7)	0.2996
T-Cho	mg/dl	188.0 (164.0–212.0)	201.0 (173.2–232.0)	< 0.0001
TG	mg/dl	101.0 (71.0–147.0)	100.0 (75.0–143.0)	0.7707
U-prot	g/day	0.54 (0.24–1.08)	0.88 (0.39–1.83)	< 0.0001
U-RBC (5 < , 5–25, 26–49, 50–99, 100 ≦)	Counts/HF	57, 186, 57, 59, 82	28, 179, 83, 60, 75	0.0045
**Histological findings**				
M0/M1		258/187	183/243	< 0.0001
E0/E1		308/137	172/254	< 0.0001
S0/S1		140/305	103/323	0.0164
T0/T1/T2		324/94/27	307/95/24	0.8934
C0/C1/C2		306/134/5	148/236/41	< 0.0001

*BMI* body mass index, *SBP* systolic blood pressure, *DBP* diastolic blood pressure, *MAP* mean arterial pressure, *TP* serum total protein, *Cr* serum creatinine, *eGFR* estimated glomerular filtration rate, *UA* serum uric acid, *T-cho* serum total cholesterol, *TG* triglyceride, *U-Prot* urinary protein excretion, *U-RBC* urinary red blood cells, *HPF* high power field, *M* mesangial hypercellularity, *E* endocapillary hypercellularity, *S* segmental sclerosis, *T* interstitial fibrosis/tubular atrophy, *C* crescents.

The Cox regression multivariate analysis indicated that lower eGFR and higher U-Prot and mean arterial pressure (MAP) were independent risk factors for progression to ESRD in all patients (Table 3). According to the Oxford classification, only the T score was an independent risk factor for progression in all patients (HR, 1.48; 95% CI, 1.10–1.99; p = 0.0085) and the treatment group (HR, 1.76; 95% CI, 1.05–2.92; p = 0.0287).

**Table 3:** Independent risk factors for progression to ESRD in the multivariate Cox regression analysis.

Baseline data	All patients	Non-treatment group	Treatment group
Clinical findings			
Age (per 10 years increase)	0.87, 0.72–1.04, P = 0.1249	0.82, 0.64–1.06, P = 0.1386	0.86, 0.63–1.16, P = 0.3320
Sex (male vs female)	1.42, 0.92–2.19, P = 0.1247	1.73, 1.03–2.90, P = 0.0394	1.30, 0.65–2.58, P = 0.4564
BMI (per 1 kg/m^2^ increase)	0.96, 0.89–1.04, P = 0.3500	1.00, 0.91–1.11, P = 0.9337	0.96, 0.84–1.08, p = 0.4851
MAP (per 10 mmHg increase)	1.30, 1.09–1.57, P = 0.0046	1.27, 0.99–1.62, P = 0.0611	1.24, 0.90–1.70, P = 0.1822
Laboratory findings			
eGFR (per 30 ml/min decrease)	2.62, 1.90–3.62, P < 0.0001	2.30, 1.48–360, P = 0.0002	3.07, 1.80–5.30, P < 0.0001
U-Prot (per 0.5 g/day increase)	1.36, 1.24–1.48, P < 0.0001	1.41, 1.25–1.60, P < 0.0001	1.46, 1.25–1.70, P < 0.0001
U-RBC (per 25/HPF increase)	0.99, 0.87–1.12, P = 0.8553	1.12, 0.98–1.30, P = 0.1525	0.86, 0.67–1.08, P = 0.2070
Histological findings			
M1 (vs. M0)	0.91, 0.62–1.34, P = 0.6432	0.84, 0.65–1.37, P = 0.4837	1.22, 0.62–2.49, P = 0.5766
E1 (vs. E0)	0.93, 0.58–1.49, P = 0.7605	0.88, 0.49–1.56, P = 0.6567	1.12, 0.48–2.71, P = 0.7974
S1 (vs. S0)	1.33, 0.80–2.19, P = 0.2667	1.75, 0.91–3.39, P = 0.0936	0.84, 0.39–2.00, P = 0.6719
T1-2 (per 1 grade increase)	1.48, 1.10–1.99, P = 0.0085	1.37, 0.92–2.02, P = 0.1183	1.76, 1.05–2.92, P = 0.0287
C1-2 (per 1 grade increase)	0.95, 0.65–1.38, P = 0.7895	1.58, 0.98–2.56 P = 0.0628	0.76, 0.36–1.52, P = 0.4505

*BMI* body mass index, *MAP* mean arterial pressure, *TP* serum total protein, *eGFR* estimated glomerular filtration rate, *U-Prot* urinary protein excretion, *U-RBC* urinary red blood cells, *M* mesangial hypercellularity, *E* endocapillary hypercellularity, *S* segmental sclerosis, *T* interstitial fibrosis/tubular atrophy, *C* crescents.

### Comparison of renal prognosis between the treatment and non-treatment group patients in the E1, S1, or S1 categories

**Figure 4:**
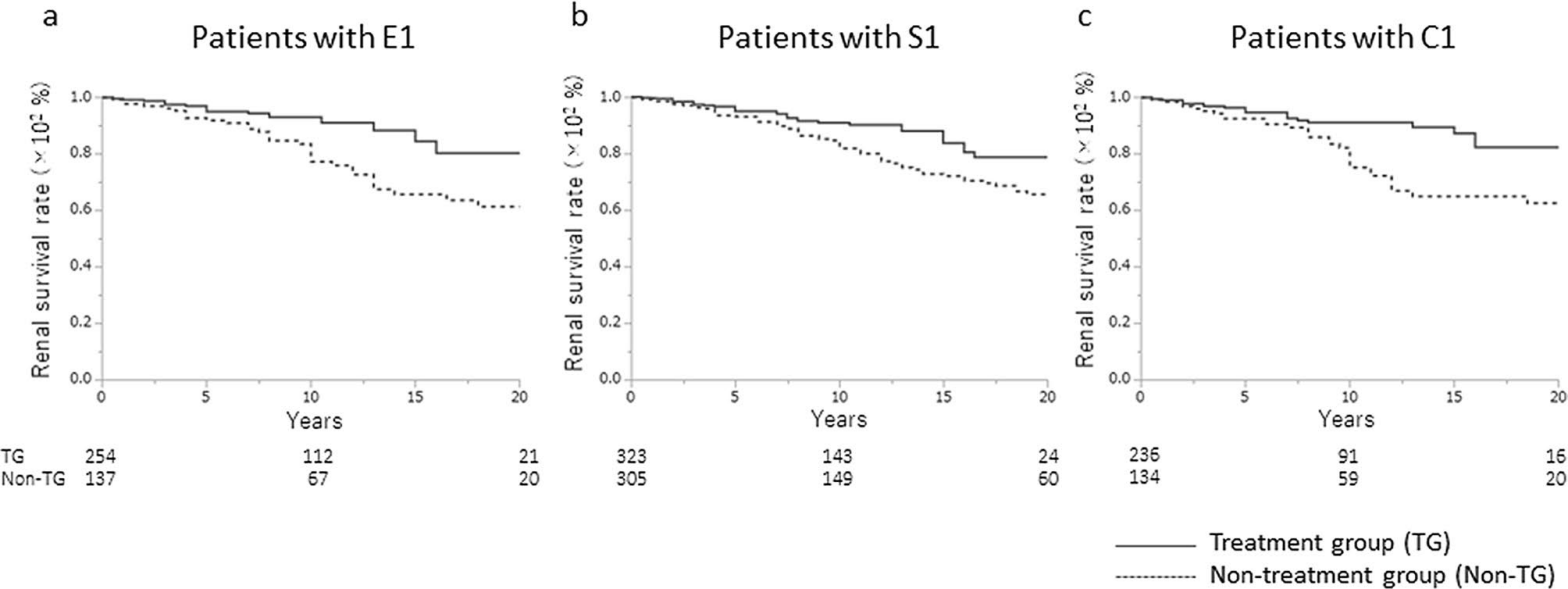
The renal survival rates for Oxford Classification E1, S1, or C1 scores. Renal survival rate in the treatment group was significantly higher than in the non-treatment group for **(a)** E1 (80.3% vs. 61.3%, p = 0.008), **(b)** S1 (78.7% versus 65.8%, p = 0.0064), and **(c)** C1 (82.5% vs. 62.7%, p = 0.0014).

Treatment with corticosteroids/immunosuppressors significantly improved the renal prognosis among IgAN patients in the E1, S1, and C1 categories compared to no treatment (Fig. 4; E1, p = 0.008; S1, p = 0.0064; and C1, p = 0.0014). In the univariate analysis (Table 4, Model 1) and the multivariate analyses considering the clinical and histological findings (Table 4, Model 2), and the clinical and histological findings and treatment (Table 4, Model 3), the use of corticosteroids/immunosuppressors decreased the risk of progression to ESRD in IgAN patients in the E1 (Model 1: HR 0.41, p = 0.0011; Model 2: HR 0.34, p = 0.0008; Model 3: HR 0.50, p = 0.0409), S1 (Model 1: HR 0.55, p = 0.0061; Model 2: HR 0.37, p < 0.0001; Model 3: HR 0.48, p = 0.0032), and C1 (Model 1: HR 0.41, p = 0.0019; Model 2: HR 0.29, p < 0.0001; Model 3: HR 0.39, p = 0.0054) categories.

**Table 4:** Univariate and multivariate Cox regression analysis of effects of corticosteroids/immunosuppressors treatment on renal prognosis, for patients with Oxford Classification E1, S1, or C1 scores.

	Model 1	Model 2	Model 3
Treatment (vs. non-treatment)			
In patients with E1 score	0.41, 0.23–0.70, P = 0.0011	0.34, 0.18–0.64, P = 0.0008	0.50, 0.26–0.97, P = 0.0409
In patients with S1 score	0.55, 0.35–0.85, P = 0.0061	0.37, 0.22–0.66, P < 0.0001	0.48, 0.29–0.78, P = 0.0032
In patients with C1 score	0.41, 0.23–0.72, P = 0.0019	0.29, 0.15–0.54, P < 0.0001	0.39, 0.20–0.76, p = 0.0054

Model 1: unadjusted; Model 2 adjusted for age, sex, body mass index, mean arterial pressure, amount of urinary protein excretion, estimated glomerular filtration rate and MEST-C score (in patients with E1 score adjusted for M, S, T, and C scores, in patients with S1 score adjusted for M, E, T, and C scores, and in patients with C1 score adjusted for M, E, S, and T scores); Model 3 adjusted for Model 2 + RAS-inhibitors and tonsillectomy.

## Discussion

This report is a validation study of the Oxford analysis of all 871 IgAN patients treated with or without corticosteroids/immunosuppressors. In our analysis, we included IgAN patients with eGFR < 30 mL/min/1.73 m^2^ and progression to ESRD within 1 year who were excluded from the original Oxford classification because it seemed important to include rapidly progressive glomerulonephritis (RPGN) and deteriorated cases at the time of renal biopsy to analyse the risk of cellular and fibrocellular crescents, which were newly included in the Oxford classification as C scores. We also included IgAN patients with U-Prot < 0.5 g/day who were excluded from the original Oxford classification because, in Japan, many IgAN patients are found in the relatively early stages of the disease by health screening checks; therefore, those mild cases were important to the analysis of IgAN diagnosed in Japan. Moreover, IgAN is generally considered a slowly progressing disease, and those mild cases should be considered in the long-term renal prognosis as in our analysis.

Salient features of our study cohort that are important to note include a relatively young age (median: 30 years old), a greater proportion of females (59.1%), well-controlled blood pressure (120/74 mmHg), relatively good renal function (eGFR 77.0 mL/min/1.73 m^2^), and mild U-Prot (0.68 g/day), including 37.0% of patients with a U-Prot level < 0.5 g/day. The distribution of Oxford classification categories, based on histological findings, were as follows: M1, 49.4%; E1, 44.9%; S1, 72.0%; T1:T2, 21.7%:5.9%; and C1:C2, 45.4%:5.3%. Almost half of the patients (48.9%) were treated using corticosteroids/immunosuppressants, with corticosteroids being used in the majority of patients (424 of 426). In our institution, corticosteroids are generally used in the treatment of IgAN for patients with higher U-Prot and U-RBC levels, stable renal function, and presence of active histological findings; these criteria are reflected in Table 2. The indications for the use of immunosuppressants in the other 13 cases included rapid disease progression, presence of comorbidities, patient’s request for a reduction of the dose and/or duration of corticosteroids or the detection of adverse effects of corticosteroid therapy, and the physician’s decision. The RAS-I was used in 33.6% of patients. This rate of use of RAS-I does not reflect the current standard treatment for IgAN. This lower than expected percentage of RAS-I use does, however, reflect the extended relevant period for our study, which included patients from as far back as 1974. The first RAS-I (captopril) was used for the treatment of hypertension in the mid-1980s in Japan. We began using RAS-I for the treatment of IgAN at our institution in the 1990s, at a rate of 19.2% up to the year 2000, with this rate having since increased to 44.5%. The lower than expected rate of RAS-I use also reflects the characteristics of our study group, with a relatively young age (median age, 31 years), larger proportion of women than men, and the majority of patients being non-hypertensive (MAP ≤ 100 mmHg, 669 (76.8%) patients, and ≤ 90 mmHg, 461 (52.9%) patients). Therefore, there was no significant indication for the use of RAS-I in our study group. We used ESRD as the endpoint of our study, although 37% of our cohort was relatively early cases of IgAN, with a U-Prot level < 0.5 g/day and slow disease progression. As such, the longer period of observation to ESRD was deemed to be more appropriate for analysis than the change in eGFR that was used for the validation of the Oxford classification for IgAN (VALIGA) in the European multicentre cohort trial^28,29^. Use of the delta eGFR was appropriate in the VALIGA study as patients in that study cohort had more severe IgAN than our cohort and, as such, eGFR is a good predictor of short-term renal survival.

In all patients, only the T score was an independent risk factor for progression to ESRD in the multivariate Cox regression analysis. In previous reports of validation studies of the Oxford classification^12–26^, the results varied because of differences in clinical backgrounds, treatments, and endpoints; however, the T score was shown to be the most important predictive factor for progression in almost of all those reports. These previous studies are in support of our results. For IgAN patients treated with corticosteroids/immunosuppressors, we found that the T score was an independent risk factor according to the multivariate Cox regression analysis. For IgAN patients without corticosteroids/immunosuppressors treatment, the E, S, T, and C scores were predictive factors for progression to ESRD according to the univariate analysis (Kaplan–Meier analysis and log-rank test). Moreover, among patients with E1, S1, or C1 lesions, renal prognosis was significantly better among those treated with than in those without corticosteroids/immunosuppressors on univariate analysis (Fig. 4 and Model 1 in Table 4) as well as on multivariate analysis considering the clinical and histological background (Model 2 in Table 4) and treatment (Model 3 in Table 4). These results are indicative of the possibility that corticosteroids/immunosuppressors can improve E1, S1, and C1 lesions. The renal survival rate of IgAN patients with a C1 score increased slightly more than that of IgAN patients with a C0 score, but it did not increase in IgAN patients with a C2 score. These results indicate that treatment with corticosteroids/immunosuppressors could improve the renal prognosis of IgAN patients with crescents in less than 25% of glomeruli, but it was difficult to improve the prognosis of IgAN patients with crescents in more than 25% of glomeruli. Recent validation studies of the Oxford classification, including the C score, showed varying results. In results from the VALIGA study^29^, for all patients (n = 1,130), the C score was not a predictive risk factor for a 50% decrease in eGFR or ESRD, and was not a predictive risk factor for the eGFR slope according to the multivariate analysis; however, for IgAN patients without corticosteroids/immunosuppressors treatment during follow-up (n = 582), the C score was an independent risk factor for the eGFR slope. These results indicated that corticosteroids/immunosuppressors improve the prognosis of IgAN patients with C scores. Furthermore, a multicentre validation study involving 3,380 IgAN patients performed in Korea indicated that C1 and C2 scores were valid predictive risk factors for progression to ESRD and decreased eGFR according to univariate and multivariate analyses^30^. In a sub-analysis of the STOP-IgAN trial involving 70 IgAN patients, for IgAN patients without corticosteroids/immunosuppressors treatment, significantly more patients with C1/C2 (38%; 3/8 patients) experienced progression to ESRD compared to patients with C0 (4%; 1/24 patients) (p = 0.0039); however, this was not true for IgAN patients treated with corticosteroids/immunosuppressors (C1/2 vs. C0: 7% [1/14 patients] vs. 13% [3/23 patients]; not significant) or for all patients (C1/2 vs. C0: 18% [4/22 patients] vs. 9% [4/47 patients]; not significant) during the 3-year analysis^31^. A validation study performed in China involving two centres with 1,152 patients showed that the 10-year renal survival rate was not significantly different among patients with C0, C1, and C2 scores, regardless of whether patients were treated with corticosteroids/immunosuppressors. However, in this study, for the patients with nephrotic syndrome, the C score was an independent factor for progression according to the multivariate analysis after adjusting for age, sex, eGFR, MAP, pathological findings, and immunosuppressors^32^. Considering all of these reports and our results, the C score is indicated to be a significant predictive factor for progression to ESRD, and the C1 score was reversible with corticosteroids/immunosuppressors, which is a good indication for this treatment.

Interestingly, our results showed that treatment with corticosteroids/immunosuppressors also improved the prognosis of IgAN patients with E1 or S1 score. Endocapillary hypercellularity (E1) was considered as the active lesion leading to the inflammation of capillaries and crescent formation. The beneficial effects of corticosteroids/immunosuppressors were seen as a reasonable result, like the previous VALIGA study^28^. When segmental sclerosis (S1) was considered as the chronic lesion, it was difficult to obtain a good response using corticosteroids/immunosuppressors. However, chronic lesions can result from continuous inflammation in the glomeruli; therefore, some IgAN patients with chronic lesions also had active lesions, and immunosuppressors improved their renal outcomes. These results were also shown in a sub-analysis of the STOP-IgAN trial^31^ and a sub-analysis^34^ of the randomized controlled trial by the IgAN study group in Japan that compared tonsillectomy combined with steroid pulse therapy and steroid pulse monotherapy^33^. In the STOP-IgAN trial, the renal survival rate for IgAN patients with T scores with progression to ESRD was improved by corticosteroids/immunosuppressors (T1/2 vs. T0: 18% [3/17 patients] vs. 7% [1/15 patients]; p = 0.0603) but not supportive therapy (T1/2 vs. T0: 33% [4/12 patients] vs. 0% [0/25 patients]; p = 0.008)^31^. A randomized controlled trial performed by the IgAN study group indicated that only the S score was an independent factor for the disappearance of both proteinuria and haematuiria by steroid pulse therapy combined with tonsillectomy; however, the M, E, and T scores were not^33,34^. These results were observed during the short-term (only 1 to 3 years); therefore, we propose that our long-term observation study shows clearer results.

This study has some limitations. First, it was performed at a single centre in Japan. Therefore, almost all of the patients in our cohort were Japanese, which means that these results might not apply to other ethnicities than Asian. Second, we did not exclude cases of deteriorated renal function and/or mild proteinuria cases at diagnosis because our aim was also to evaluate the rapid progressive cases (cases of deteriorated renal function), which seem to have more crescent formation, and also evaluate early-stage cases (mild proteinuria cases) especially those diagnosed in Japan. We have shown the cohort results when applying the inclusion criteria stated in the original Oxford classification (Supplemental Materials). However, the results were clearer in the cohort where our criteria were applied than in the cohort with the criteria of the original Oxford classification. In Japan, IgAN was mainly found in health screening checks, and the criterion for biopsy was the early stage of the disease; therefore, the evaluation criteria of the Oxford classification might be different. Specifically, although delta eGFR might be the most appropriate marker to evaluate the short-term renal prognosis among patients with more than mild IgAN, the Oxford Classification was useful in our study, in which we included cases with a wide range of IgAN disease severity, from mild (slowly progressing) to deteriorating IgAN. Third, this study was a retrospective cohort analysis. To establish strong evidence, large multicentre prospective control trials, including patients of other races, should be performed.

## Conclusions

In this study, we report a validation of the Oxford analysis and found that the T score was the most important predictive factor of renal survival in all IgAN patients despite treatment with corticosteroids/immunosuppressors. However, corticosteroids/immunosuppressors improved the long-term renal prognosis for IgAN patients with E1, S1, and C1 scores.

## Methods

### Study population and study design

In this study, 1,147 primary IgAN patients were diagnosed via renal biopsy at Tokyo Women’s Medical University between 1974 and 2015. In those patients, 871 patients had more than eight glomeruli according to the renal biopsy. They were observed for at least 1 year after renal biopsy unless ESRD occurred within 1 year and were not diagnosed with a systemic disease, such as systemic lupus erythematosus, liver cirrhosis, and IgA vasculitis with nephritis. The patients with eGFR < 30 mL/min/1.73 m^2^ and/or proteinuria < 0.5 g/day were not excluded from evaluating the rapid progressive cases at diagnosis and mild cases. Of those 871 patients, 426 patients began treatment with corticosteroids/immunosuppressors within 1 year after renal biopsy as the initial treatment (treatment group), and 445 patients did not (non-treatment group). Validation of the Oxford classification was determined using all patients, and comparisons were made between the two groups.

This retrospective cohort study was conducted in accordance with the guidelines of the Declaration of Helsinki and was approved by the Medical Ethics Committee of Tokyo Women’s Medical University (reference #5104). Written informed consent to perform a renal biopsy was obtained from all patients; patients were able to opt-out of this study by visiting our institution’s website.

### Diagnosis of IgAN and histological evaluation of renal biopsy specimens

The indication for renal biopsy generally depended on a higher amount of proteinuria (> 1.0 g/day), U-RBC (> 50/HPF), stable renal function (chronic kidney disease (CKD) grade 1 or 2), and/or severe histological active lesions, such as endocapillary hypercellularity and cellular and fibro-cellular crescents. The criteria for renal biopsy included rapid progressive glomerulonephritis, with deteriorating renal function, and patients’ background and assessment/treatment goals in cases with mild urinary and/or histological findings.

All renal biopsy specimens were obtained using a percutaneous needle biopsy. Specimens were fixed in 10% phosphate-buffered formalin (pH 7.2), embedded in paraffin, and cut into 4-μm-thick sections. The sections were stained with haematoxylin and eosin, periodic acid–Schiff, silver methenamine, and Masson trichrome; then, they were examined by light microscopy. For the immunofluorescence analysis, the specimens were fixed with cold acetone, and frozen sections were routinely subjected to fluorescence by IgG, IgA, IgM, C3, C4, C1q, fibrinogen, and fibronectin. IgAN was diagnosed based on mesangial proliferative changes in light microscopic findings, mesangial IgA and C3 deposition in immunofluorescence findings, and mesangial electron-dense deposits in electron microscopic findings.

Histological findings were graded according to the Oxford classification^10,11,27^.

### Clinical and laboratory data

Patient sex, age, body mass index (BMI), SBP, DBP, MAP, and duration of the observation period were recorded. Laboratory data included serum TP, creatinine (Cr), eGFR, uric acid (UA), total cholesterol (T-Cho), triglycerides, U-Prot, and urinary red blood cells (U-RBC) at the time of renal biopsy; these were evaluated as baseline data. The eGFR was calculated using the modified isotope dilution mass spectrometry-modification of diet in renal disease (IDMS-MDRD) study for Japanese individuals (eGFR = 194 × S-Cre^-1.094^ × age^-0.287^ × 0.739 [if female])^35^. Time to progression to ESRD, defined as requiring dialysis or renal transplantation, was evaluated as the endpoint, and the risk factors associated with progression to ESRD were evaluated.

### Statistical analysis

Data were expressed as mean ± standard deviation (SD) for normally distributed data and as the median and interquartile range (IQR) for skewed data. Cumulative renal survival rates until ESRD were calculated according to the Kaplan–Meier method and compared using the log-rank test. The unpaired Student’s t-test for normally distributed data and Mann–Whitney’s U test for skewed data were used to compare the clinical findings of patients treated with or without corticosteroids/immunosuppressors. The chi-squared test was used to compare the sex distribution, the number of patients with each grade of U-RBC at the time of renal biopsy, and the Oxford classification of groups treated with or without corticosteroids/immunosuppressors. Univariate and multivariate Cox regression analyses were performed to evaluate the risk of deterioration to ESRD. The univariate analyses indicated that sex (male/female) and Oxford classification were categorical variables, and that age, BMI, MAP, eGFR, UA, T-Cho, U-Prot, and U-RBC were quantitative variables. The results of these univariate and multivariate analyses are expressed as hazard ratios (HRs) with 95% confidence intervals (CIs). In all analyses, *p* < 0.05 was considered statistically significant. All analyses were performed using JMP Pro 13.0.0 (SAS Institute Inc., Cary, NC, USA).

## Supplementary information

Supplementary information

## Data Availability

The datasets during and/or analysed during the current study available from the corresponding author on reasonable request.
