# Supplementary material for: Validation of the revised Oxford classification for IgA nephropathy considering treatment with corticosteroids/immunosuppressors
Source: Sci Rep. 2020 Jul 7;10:11151. doi: 10.1038/s41598-020-68087-y (PMC7341848; doi:10.1038/s41598-020-68087-y)
Supplement: Supplementary file 1 — Supplementary information [file 41598_2020_68087_MOESM1_ESM.pdf]

Validation of the revised Oxford classification for IgA nephropathy considering treatment with corticosteroids/immunosuppressors

Takahito Moriyama*, Kazunori Karasawa, Yoei Miyabe,
Kenichi Akiyama, Shota Ogura, Tomo Takabe, Naoko Sugiura,
Momoko Seki, Yuko Iwabuchi, Keiko Uchida, Kosaku Nitta

Affiliations: Department of Nephrology, Tokyo Women's Medical University, Tokyo, Japan

Supplementary Figure S1. Flow diagram of patient recruitment and enrollment.

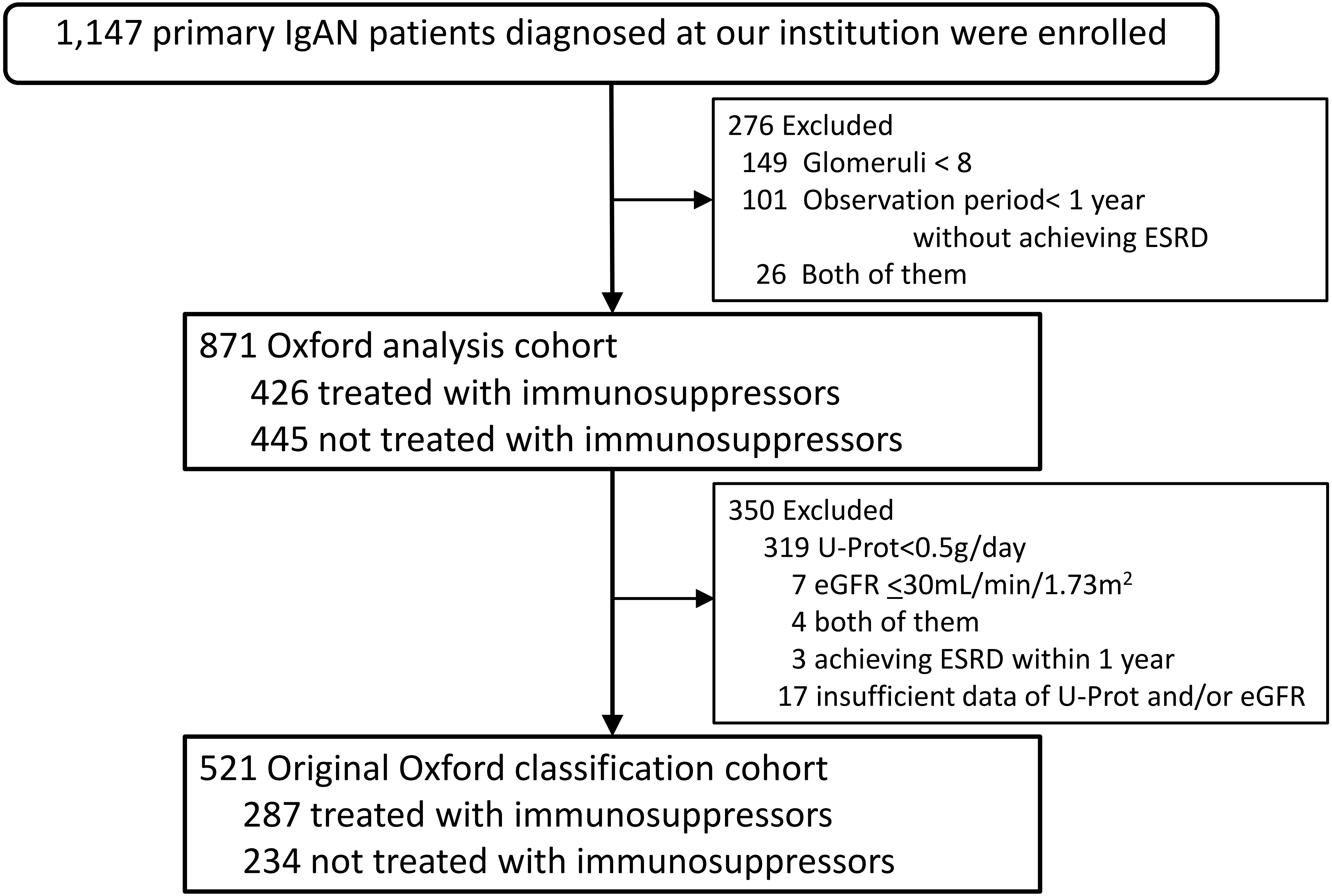

Supplementary Table S1. Comparison of baseline characteristics among all patients, non-treatment, and treatment groups

Baseline data	All patients	Non-treatment group	Treatment group	P-value between non-treatment and treatment groups
Clinical findings				
**Age (years)**	32.0 (25.3-43.0)	33.0 (26.0-44.0)	31.0 (25.0-41.0)	0.8983
**Sex (Male /female)**	293/227	123/94	170/133	0.8960
**BMI (kg/m^2)**	21.5 (19.8-23.7)	21.6 (19.7-23.5)	21.5 (19.9-23.9)	0.9282
**SBP (mmHg)**	122.0 (112.0-136.0)	124.0 (115.0-139.3)	120.0 (111.0-132.0)	0.0056
**DBP (mmHg)**	77.0 (68.0-85.0)	79.0 (70.0-86.3)	76.0 (67.0-83.0)	0.0986
**MAP (mmHg)**	92.0 (83.3-101.0)	93.0 (84.4-103.6)	90.7 (82.7-100.0)	0.0268
Laboratory findings				
**TP (g/dL)**				
**Cr (mg/dl)**	6.6 (6.1-7.0)	6.7 (6.1-7.1)	6.6 (6.1-7.0)	0.0845
**eGFR (ml/min/1.73m^2)**	0.84 (0.69-1.08)	0.88 (0.69-1.18)	0.81 (0.68-1.02)	0.0092
**UA(mg/dl)**	71.8 (56.2-90.3)	66.8 (52.6-85.6)	75.2 (57.1-94.0)	0.0098
**T-Cho (mg/dl)**	5.7 (4.8-6.8)	5.8 (4.8-7.2)	5.7 (4.8-6.7)	0.3448
**TG (mg/dl)**	202.9 (173.5-234.0)	192.0 (170.5-223.5)	209.0 (176.5-239.5)	0.0024
**U-prot (mg/dl)**	107.5 (78.8-164.3)	111.5 (78.0-166.3)	106.0 (79.0-156.8)	0.3526
**U-RBC (counts/HF) (5<, 5-25, 26-49, 50-99,100≦)**	1.17 (0.74-1.95)	1.00 (0.69-1.51)	1.3 (0.8-2.26)	0.0002
	47, 201, 90, 63, 108	26, 85, 35, 24, 46	21, 116, 55, 48, 62	0.1988
Histological findings				
**M0/M1**	228/292	111/106	117/186	0.0045
**E0/E1**	256/263	145/72	112/191	<0.0001
**S0/S1**	106/414	47/170	59/244	0.5424
**T0/T1/T2**	326/156/38	128/70/19	198/86/19	0.2860
**C0/C1/C2**	235/241/43	142/71/4	94/170/39	<0.0001

Abbreviations; BMI: Body mass index, SBP: systolic blood pressure, DBP: diastolic blood pressure, MAP mean arterial pressure, TP: serum total protein, Cr: serum creatinine, eGFR: estimated glomerular filtration rate, UA: serum uric acid, T-cho: serum total cholesterol, TG: triglyceride, U-Prot: urinary protein excretion, U-RBC: urinary red blood cells, HPF: high power field, M: mesangial hypercellularity, E: endocapillary hypercellularity, S: segmental sclerosis, T: interstitial fibrosis/tubular atrophy, C: crescents,

Supplementary Figure S2. Renal survival rates of all patients in each Oxford classification

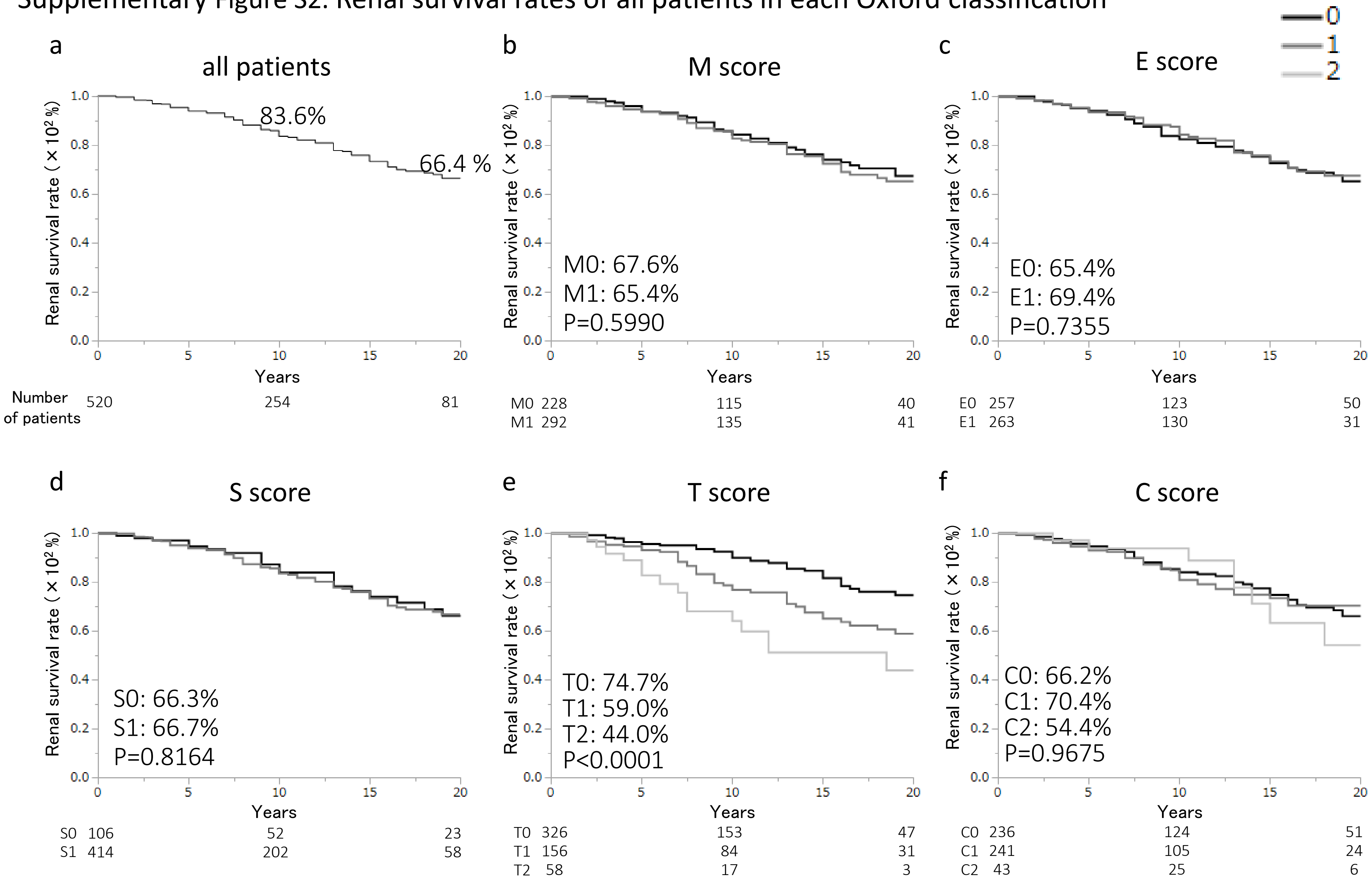

Supplementary Figure S3. Renal survival rates of the non-treatment group in each Oxford classification

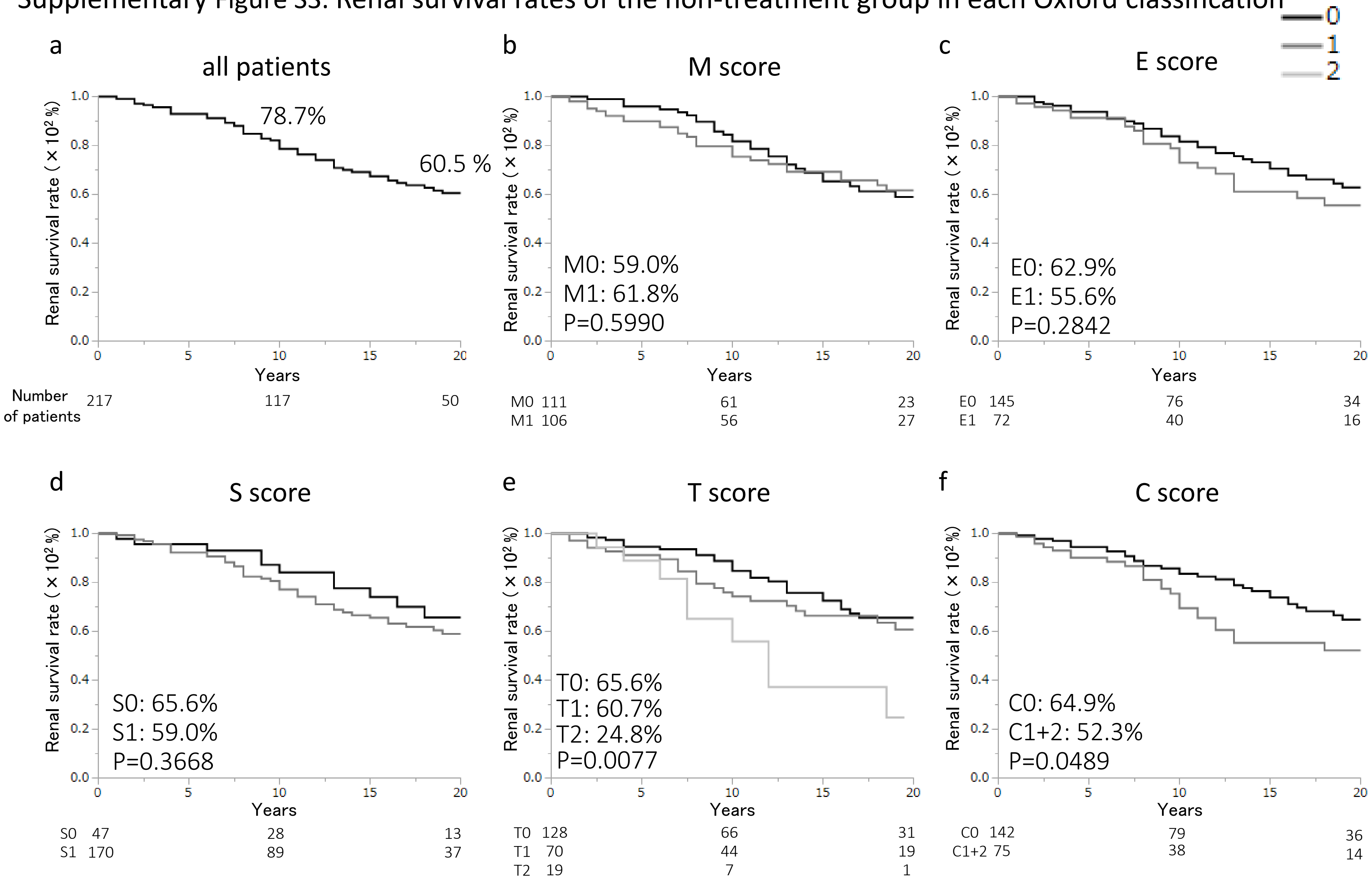

Supplementary Figure S4. Renal survival rates of the treatment group in each Oxford classification

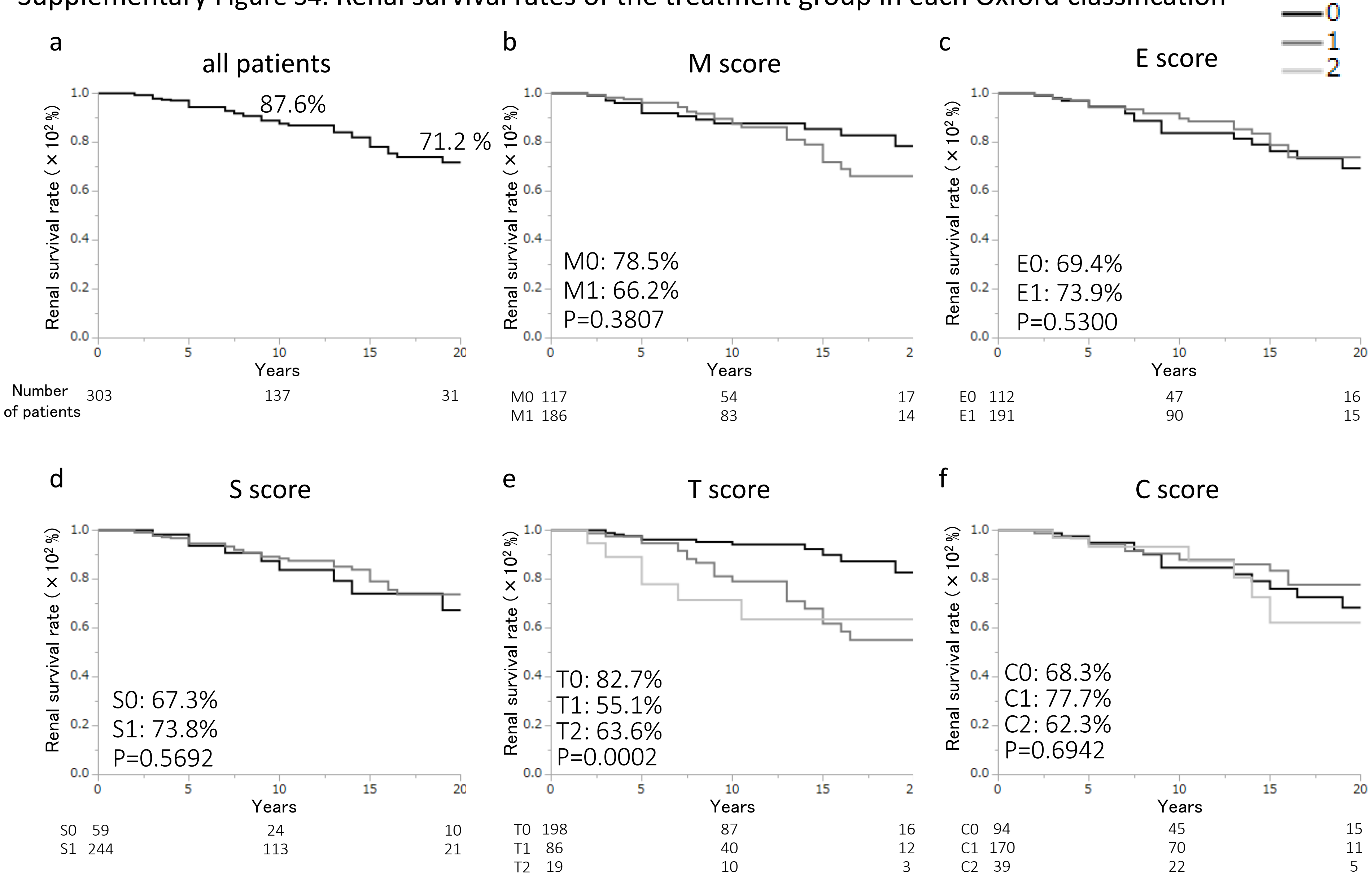

Supplementary Table S2. Independent risk factors for progression to ESRD in the multivariate Cox regression analysis in each group

Baseline data	All patients	Non-treatment group	Treatment group
Clinical findings			
**Age** (per 10 years increase)	0.79, 0.54-1.14, P=0.2106	0.69, 0.39-1.19, P=0.1797	0.82, 0.46-1.44, P=0.4921
**Sex** (male vs female)	1.65, 1.07-2.57, P=0.1565	1.76, 0.95-3.37, P=0.0744	1.50, 0.75-2.98, P=0.2461
**BMI** (per 1kg/m^2 increase)	0.89, 0.76-1.04, P=0.3500	0.98, 0.79-1.21, P=0.8281	0.85, 0.66-1.09, p=0.2032
**MAP** (per 10 mmHg increase)	1.29, 1.06-1.57, P=0.0117	1.30, 0.98-1.73, P=0.0724	1.30, 0.94-1.77, P=0.1077
Laboratory findings			
**eGFR** (per 30ml/min decrease)	2.39, 1.67-3.49, P<0.0001	2.29, 1.38-3.95, P=0.0011	2.26, 1.27-4.10, P=0.0052
**U-Prot** (per 0.5 g/day increase)	1.74, 1.42-2.12, P<0.0001	1.70, 1.29-2.24, P=0.0002	2.00, 1.44-2.82, P<0.0001
**U-RBC** (per 25/HPF increase)	1.74, 0.87-1.12, P=0.8542	1.13, 0.96-1.32, P=0.1319	0.86, 0.68-1.08, P=0.2076
Histological findings			
**M1** (vs. M0)	0.98, 0.64-1.49, P=0.8947	1.01, 0.58-1.77, P=0.9764	1.32, 0.65-2.80, P=0.3388
**E1** (vs. E0)	0.92, 0.54-1.54, P=0.7484	0.91, 0.45-1.80, P=0.7969	1.12, 0.46-2.81, P=0.7999
**S1** (vs. S0)	1.27, 0.75-2.24, P=0.3787	2.22, 1.05-5.14, P=0.0365	0.82, 0.38-1.97, P=0.6458
**T1-2** (per 1 grade increase)	1.13, 0.81-1.56, P=0.4735	0.95, 0.58-1.52, P=0.8288	1.38, 0.80-2.37, P=0.2387
**C1-2** (per 1 grade increase)	0.96, 0.64-1.44, P=0.8496	1.52, 0.86-2.66 P=0.1467	0.73, 0.35-1.46, P=0.3830

Abbreviations; BMI: Body mass index, MAP mean arterial pressure, TP: serum total protein, eGFR: estimated glomerular filtration rate, U-Prot: urinary protein excretion, U-RBC: urinary red blood cells, M: mesangial hypercellularity, E: endocapillary hypercellularity, S: segmental sclerosis, T: interstitial fibrosis/tubular atrophy, C: crescents,
